# Metabolomic stratification of shock: pathophysiological insights for personalized critical care

**DOI:** 10.1186/s13613-025-01532-1

**Published:** 2025-07-31

**Authors:** Frederic Sangla, Karim Bendjelid, Federico Aletti, Vicente Ribas, Antoine Herpain, Bernardo Bollen Pinto, David Legouis

**Affiliations:** 1 Intensive Care Unit, Department of Anesthesiology, Pharmacology, Critical Care and Emergency Medicine, University Hospital of Geneva, 1205 Geneva, Switzerland; 2 Faculdade Israeilita Albert Einstein, Sao Paulo, Brazil; 3 Eurecat, Technology Centre of Catalonia, Digital Health, Barcelona, Spain; 4 Experimental Laboratory of Intensive Care, Erasme University Hospital, Hôpitaux Universitaires de Bruxelles, Université Libre de Bruxelles, Brussels, Belgium; 5 Intensive Care Department, Saint-Pierre University Hospital, Université Libre de Bruxelles, Brussels, Belgium; 6 Division of Anesthesiology, Department of Anesthesiology, Pharmacology, Critical Care and Emergency Medicine, University Hospital of Geneva, Geneva, Switzerland; 7 Laboratory of Nephrology, Department of Physiology and Cell Metabolism, University of Geneva, Geneva, Switzerland

**Keywords:** 55 in-hospital mortality, Septic shock, Mass spectrometry, Metabolomics, Cluster analysis

## Abstract

**Background:**

Shock, encompassing septic and cardiogenic etiologies, is a life-threatening condition associated with systemic inflammation, metabolic dysregulation, and high mortality in intensive care units. Traditional clinical markers often fail to capture the complexity of this syndrome, limiting personalized therapeutic approaches. Advances in metabolomics enable comprehensive analysis of metabolic disruptions, providing novel insights into shock pathophysiology. This study aimed to cluster critically ill patients with shock into metabolic phenotypes and investigate their associations with clinical severity.

**Results:**

We analyzed metabolomic profiles from 60 critically ill patients with shock at ICU admission using Uniform Manifold Approximation and Projection (UMAP) for dimensionality reduction and Density-Based Spatial Clustering of Applications with Noise (DBSCAN) for clustering. Three distinct clusters were identified: Cluster 1 (n = 13) exhibited the highest severity (median APACHE II: 29) and mortality (54%), with elevated biogenic amines, sugars, and sphingolipids, reflecting intense metabolic activation. Cluster 2 (n = 24), despite having low initial severity (median APACHE II: 25), demonstrated high mortality (38%) and was characterized by elevated glycerophospholipids and sphingolipids as in cluster 1, without enhanced biogenic amines and sugars, indicating inadaptive metabolic responses. Cluster 3 (n = 23) showed the lowest severity (median APACHE II: 22) and mortality (9%), with uniformly reduced metabolite levels, suggesting an adaptive metabolic profile.

**Conclusions:**

Shock patients exhibit distinct metabolic phenotypes associated with clinical severity and outcomes. Metabolomic profiling offers a promising avenue for precision medicine in critical care by uncovering pathophysiological insights. Future research should validate these findings, identify practical biomarkers, and explore therapeutic interventions tailored to specific metabolic profiles.

**Supplementary Information:**

The online version contains supplementary material available at 10.1186/s13613-025-01532-1.

## Background

Shock remains the main reason for ICU admission and the leading cause of morbidity and mortality in critically ill patients, despite advancements in intensive care medicine [1]. This life-threatening condition arises from diverse underlying mechanisms but is universally characterized by systemic inflammation, hemodynamic instability, and profound metabolic disturbances. The heterogeneous nature of shock complicates its clinical management, as traditional diagnostic and prognostic markers often fail to account for patient-specific variations [2]. Consequently, personalized therapeutic strategies are urgently needed to improve outcomes.

Metabolomics, a high-throughput analytical approach that comprehensively profiles small molecules involved in metabolic processes, has emerged as a powerful tool for understanding the complex pathophysiology of critical illnesses [3, 4]. By identifying distinct metabolic signatures, metabolomics offers novel insights into disease mechanisms and enables the stratification of patients into clinically meaningful subgroups. This stratification could serve as the foundation for precision medicine, tailoring interventions to the unique metabolic profiles of patients [5]. Recent advancements in dimensionality reduction techniques, such as Uniform Manifold Approximation and Projection (UMAP), and clustering algorithms, such as Density-Based Spatial Clustering of Applications with Noise (DBSCAN), have enabled the effective categorization of complex metabolomic data [6–8]. These tools facilitate the identification of patient clusters with shared metabolic and clinical characteristics, shedding light on the interplay between metabolic adaptations and maladaptations in critical care scenarios [9].

In this study, we applied metabolomic profiling and advanced clustering methods to critically ill patients with shock at ICU admission. Our primary aim was to identify distinct metabolic phenotypes and explore their associations with clinical severity and outcomes. By integrating metabolomic insights with clinical data, this research seeks to deepen our understanding of shock pathophysiology and advance the development of personalized management strategies in critical care.

## Methods

### Patients

This study is an ancillary investigation of the prospective observational trial ShockOmics, a European research project titled “Multiscale approach to the identification of molecular biomarkers in acute heart failure induced by shock” (Nr. 602706—ClinicalTrials.gov Identifier NCT02141607) [10]. The protocol details are described by Aletti et al. [11].

Patients admitted with either cardiogenic or septic shock to the ICUs of Geneva University Hospitals (Geneva, Switzerland) and Erasme University Hospital (Brussels, Belgium) between October 2014 and March 2016 were screened for inclusion.

Inclusion criteria were patients diagnosed with septic or cardiogenic shock, older than 18 years, with a Sequential Organ Failure Assessment (SOFA) score greater than 5, arterial lactate levels of 2 mmol/l or higher, and a documented source of infection at admission for septic shock patients.

Septic shock was defined as sepsis-induced hypotension, characterized by systolic blood pressure < 90 mmHg or a drop of > 40 mmHg from baseline, or mean arterial pressure < 65 mmHg, persisting despite adequate fluid resuscitation. Cardiogenic shock (CS) was defined as hypotension (systolic blood pressure < 90 mmHg or a drop of > 40 mmHg from baseline or mean arterial pressure < 70 mmHg) persisting despite adequate fluid resuscitation, with a Cardiac Index < 1.8 L/min/m^2^ without support or < 2.0–2.2 L/min/m^2^ with inotropic drugs or cardiac assistance devices.

Exclusion criteria included patients with a high risk of death within the first 24 h after admission, immunosuppression, hematological diseases, metastatic malignancy, preexisting dialysis, decompensated cirrhosis, or those who had received more than 4 units of red blood cells or any fresh frozen plasma before screening.

### Data collection

Data on demographics, clinical and laboratory variables were recorded at ICU admission. Data on ICU and hospital stay, and data on mortality were extracted from medical records.

Metabolites dosages were performed within 16 h of ICU admission in collected plasma sample. Patterns of shocked patients were defined according to their metabolomic profile.

### Identification of metabolites

Targeted metabolomic profiling was performed using the AbsoluteIDQ® p180 kit (Biocrates, Innsbruck, Austria), which enables the absolute quantification of 186 metabolites, including 21 amino acids, 19 biogenic amines, 40 acylcarnitines and carnitine, 90 glycerophospholipids, 15 sphingolipids, and 1 monosaccharide. Details regarding the methodology and data preprocessing have been comprehensively described in previous studies [12, 13]. Briefly, this validated platform combines flow injection analysis and liquid chromatography tandem mass spectrometry (LC–MS/MS). Detection was performed in multiple reaction monitoring (MRM) mode using isotope-labeled internal standards. This method, which requires a low sample volume (10 µL), has demonstrated high inter-laboratory reproducibility (> 85% of metabolites with coefficients of variation < 20% when benchmarked against the NIST SRM 1950 reference plasma) [14], and is widely applied in critical care and multi-omics studies [15].

To ensure data quality and analytical robustness, metabolites were excluded from downstream analyses if they met either of the following criteria: (1) > 20% missing values within any patient cluster, or (2) detectable concentrations in < 50% of all samples (i.e., below the lower limit of detection in > 50%). Based on these criteria, 130 of the 186 measurable metabolites were retained for statistical analysis. The full list of measurable metabolites, their biochemical classes and whether they were retained for analysis based on quality control, is provided in Supplementary Table 1.

### Main outcome

Overall in-hospital mortality was compared across identified clusters.

### Statistical analyses

Baseline characteristics were described as median with interquartile ranges (25th–75th percentiles) for continuous variables and as absolute and relative frequencies (%) for categorical variables. Group comparisons were conducted using Kruskal–Wallis tests for continuous variables and Fisher’s exact tests for categorical variables. Statistical significance was defined as a p-value of < 0.05. All analyses were performed using R software.

For mortality analyses, covariates associated with in-hospital death (p < 0.1) in univariate logistic regression were considered for inclusion in the multivariable model. Variables were further selected using backward elimination based on statistical significance (p < 0.05) and consideration of model parsimony, given the limited number of events (n = 18). To minimize overfitting, the final model was restricted to three predictors across four degrees of freedom: cluster membership (categorical, 2 d.f.), number of organ failures at admission (continuous), and APACHE II score (continuous), resulting in an event-per-variable (EPV) ratio of 4.5.

Continuous variables were standardized (z-score transformation) prior to model fitting to improve the interpretability of effect sizes and enhance numerical stability [16]. Internal validation of the final model was performed using bootstrap resampling with 1,000 iterations, following the approach recommended by Austin and Steyerberg for small-sample logistic regression models [17]. Model performance was assessed using optimism-corrected discrimination (c-index), calibration slope, maximum calibration error (Emax), and shrinkage-consistent effect attenuation (g-index).

### Unsupervised clustering

The complete matrix of metabolite concentrations across patients was used as input for Uniform Manifold Approximation and Projection (UMAP), using the default settings of the *umap* R package to project the data into a two-dimensional space. The resulting embeddings were then clustered using the Density-Based Spatial Clustering of Applications with Noise (DBSCAN) algorithm, implemented via the *dbscan* R package.

We set the minPts parameter to the default value of 5, in line with standard recommendations (i.e., at least the number of dimensions plus one; here, 2 + 1 = 3). The eps parameter was empirically determined using a k-distance plot (with k = 4, i.e., minPts − 1), which showed a clear inflection point at approximately 0.7. We therefore selected eps = 0.7 as the final value, which yielded a stable and interpretable three-cluster solution with no noise points.

To assess the stability and reproducibility of the clustering results, we performed a bootstrap resampling procedure with 1000 iterations. For each iteration, patients were randomly sampled with replacement, and the same UMAP + DBSCAN pipeline was applied using fixed parameters (eps = 0.7, minPts = 5). Cluster assignments from each iteration were compared to the original clustering using the Jaccard similarity index, calculated over patients common to both the bootstrap and original sets.

### Sensitivity Analysis

To evaluate the robustness of the unsupervised clustering results, we performed a sensitivity analysis aimed at identifying and excluding patients with extreme metabolomic profiles. For each patient, we computed the maximum absolute Z-score across all measured metabolites. Patients with at least one metabolite exhibiting a Z-score greater than 5 were classified as outliers and excluded from the dataset. The dimensionality reduction (UMAP) and clustering (DBSCAN) procedures were then repeated on the reduced cohort of remaining patients, using the same parameters as in the primary analysis (eps = 0.7, minPts = 5).

To assess the consistency of cluster assignments before and after outlier exclusion, we generated an alluvial plot comparing individual cluster memberships between the original and sensitivity analyses. This allowed visual evaluation of cluster stability and potential shifts in classification attributable to extreme profiles.

## Results

From October 2014 to March 2016, 60 critically ill patients enrolled in the ShockOmics European research project were analyzed based on metabolomic profiles obtained at ICU admission. The dataset, comprising 130 metabolites, underwent dimensionality reduction using Uniform Manifold Approximation and Projection (UMAP) and clustering via the Density-Based Spatial Clustering of Applications with Noise (DBSCAN).

**Fig. 1:**
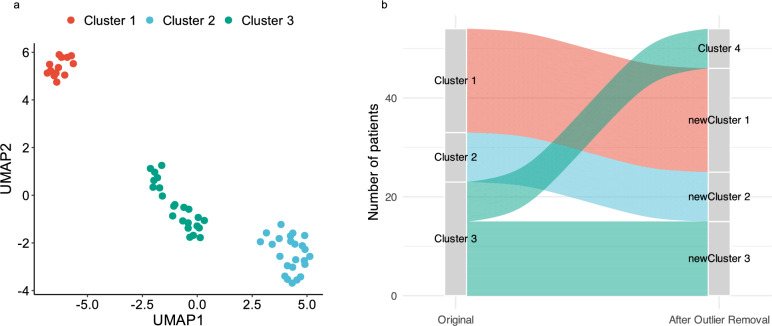
Clustering of Patients Based on Metabolomic Profiles (**a**) UMAP projection of the metabolomic profiles of patients at ICU admission, revealing three distinct clusters identified by DBSCAN. **b** Alluvial plot illustrating the stability of cluster assignments after exclusion of six patients with extreme metabolomic values (Z-score > 5)

Three distinct patient clusters were identified: Cluster 1 (13 patients), Cluster 2 (24 patients), and Cluster 3 (23 patients) (Fig. 1a). Cluster assignments were highly stable across 1000 bootstrap iterations (mean Jaccard index = 0.964; see Supplementary Fig. 1). Baseline characteristics for each cluster are summarized in Table 1, and detailed pairwise p-values for all variables are provided in Supplementary Table 2. Patients in Cluster 1 presented with more severe clinical indicators, such as higher lactate levels, lower pH, and elevated APACHE II scores. By contrast, baseline characteristics of Clusters 2 and 3 were largely similar (Supplementary Fig. 2). To further assess the robustness of the clustering results, we conducted a sensitivity analysis excluding six patients with extreme metabolomic profiles, defined as having a Z-score > 5 for any metabolite. The UMAP and DBSCAN clustering was reapplied to the reduced dataset of 54 patients using the same parameters as in the primary analysis. Cluster assignments remained unchanged for Clusters 1 and 2, confirming the stability of these phenotypes. In contrast, Cluster 3 fragmented into two smaller subgroups (Fig. 1b), suggesting greater internal heterogeneity among these lower-risk patients. These findings support the overall robustness of the clustering approach while highlighting potential metabolic diversity within Cluster 3.

**Table 1:** Baseline characteristics 178 and outcome of the 60 included patients

	Cluster 1 (*n* = 13)	Cluster 2 (*n* = 24)	Cluster 3 (*n* = 23)	Total (*n* = 60)	*p value*
Admission cause					
Septic	7 (54)	17 (71)	15 (65)	39 (65)	0.61
Cardiovascular	6 (46)	7 (29)	8 (35)	21 (35)	
Demographics and underlying diseases					
Sex, male, n (%)	8 (62)	17 (71)	19 (83)	44 (73)	0.39
Age, mean (IQR)	69 (58–93)	62 (27–91)	69 (24–86)	66 (24–93)	0.47
Arterial hypertension, n (%)	9 (69)	12 (50)	13 (57)	34 (57)	0.53
Diabetes Mellitus, n (%)	7 (54)	0	11 (48)	18 (30)	< 0.01
Coronary artery disease, n (%)	2 (15)	6 (25)	6 (26)	14 (23)	0.86
Other cardiovascular disease, n (%)	3 (23)	3 (13)	5 (22)	11 (18)	0.67
Chronic lung disease, n (%)	4 (31)	5 (21)	3 (13)	12 (20)	0.42
Chronic kidney disease, n (%)	1 (8)	5 (21)	2 (9)	8 (13)	0.49
Chronic liver failure, n (%)	0.0	3 (13)	2 (9)	5 (8)	0.62
Characteristics at ICU Admission					
Anuria and/or RRT, n (%)	1 (8)	2 (8)	0	3 (5)	0.43
Systolic arterial blood pressure (mmHg), mean (IQR)	86 (60–112)	87 (61–125)	88 (66–123)	87 (60–125)	0.94
Diastolic arterial blood pressure (mmHg), mean (IQR)	47 (39–61)	46 (30–63)	48 (30–67)	47 (30–67)	0.81
Mean arterial blood pressure (mmHg), mean (IQR)	59 (49–69)	60 (47–83)	61 (42–84)	60 (42–84)	0.81
Heart rate (bpm), mean (IQR)	106 (59–137)	103 (46–145)	107 (52–177)	106 (46–177)	0.90
Creatinine (mg/dL), mean (IQR)	2.02 (0.8–5.3)	1.66 (0.6–4.8)	1.67 (0.7–5.9)	1.74 (0.6–5.9)	0.34
Na^+^ (mmol/L), mean (IQR)	137 (120–145)	139 (133–151)	139 (125–149)	139 (120–151)	0.98
K^+^ (mmol/L), mean (IQR)	4.3 (2.6–5.7)	4.4 (3.3–6.2)	4.1 (1.5–5.9)	4.2 (1.5–6.2)	0.64
Cl^−^ (mmol/L), mean (IQR)	101 (87–110)	105 (92–116)	104 (91–121)	104 (87–121)	0.34
Lactate, mean (IQR)	8.6 (3.4–15.0)	5.0 (1.2–11.9)	4.4 (1.1–14.7)	5.5 (1.1–15.0)	< 0.01
pH, mean (IQR)	7.2 (6.8–7.5)	7.3 (7.0–7.5)	7.3 (6.8–7.5)	7.3 (6.8–7.5)	0.03
PaO2 (mmHg), mean (IQR)	105 (47–216)	99 (50–239)	81 (53–117)	94 (47–239)	0.17
PaCO2 (mmHg), mean (IQR)	46 (28–72)	42 (22–79)	37 (25–72)	41 (22–79)	0.04
HCO3^−^ (mmol/L), mean (IQR)	18 (10–29)	18 (9–28)	19 (10–25)	18 (9–29)	0.81
Base Excess (mmol/L), mean (IQR)	− 12 (− 27–5)	− 7 (− 21–3)	− 8 (− 22–2)	− 8 (− 27–2)	0.18
SOFA score, mean (IQR)	13 (8–17)	13 (6–17)	1 1 (3–17)	12 (3–17)	0.05
APACHE II score, mean (IQR)	29 (18–41)	25 (13–36)	22 (7–37)	25 (7–41)	0.04
Outcomes					
ICU stay (d), mean (IQR)	8 (1–27)	9 (1–23)	6 (1–18)	8 (1–27)	0.54
Hospital stay (d), mean (IQR)	24 (2–74)	19 (2–59)	25 (3–71)	22 (2–74)	0.60
ICU mortality, n (%)	5 (39)	8 (33)	2 (9)	15 (25)	0.07
Hospital mortality, n (%)	7 (54)	9 (38)	2 (9)	18 (30)	< 0.01

Data are presented as mean (percentage) or as median (interquartile range)

180 IQR = Interquartile range

181 SOFA = Sepsis-related organ failure assessment

182 APACHE II = Acute Physiology And Chronic Health Evaluation II

**Fig. 2:**
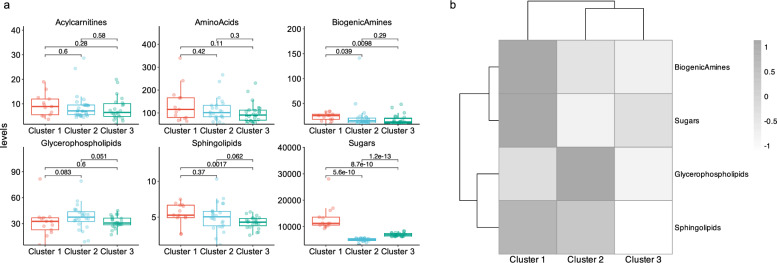
Metabolite Levels and Clusters (**a**) Levels of metabolite families across clusters and (**b**) Heatmaps showing the median levels of differentially expressed metabolite families across clusters

Metabolite levels varied significantly among the clusters. Specifically, biogenic amines and sugars were elevated in Cluster 1 compared to Clusters 2 and 3 (p = 0.01 and p < 0.01 respectively). Sphingolipids were higher in Clusters 1 and 2 relative to Cluster 3 (p = 0.006). Glycerophospholipids were predominantly elevated in Cluster 2 compared to Clusters 1 and 3 (p = 0.03) (Fig. 2a).

These findings indicate distinct metabolic profiles for each cluster: Cluster 1 is characterized by high levels of biogenic amines and sugars, Cluster 2 by elevated glycerophospholipids, and Cluster 3 by comparatively lower levels across all analyzed metabolite families (Fig. 2b).

**Fig. 3:**
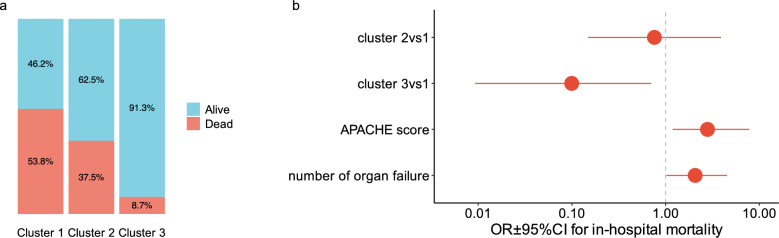
In-Hospital Mortality and Predictors (**a**) Distribution of in-hospital mortality rates across clusters and (**b**) Forest plot showing odds ratios (OR) and 95% confidence intervals (CI) from a multivariate logistic regression model predicting in-hospital mortality. Covariates include metabolic phenotype membership, APACHE II score, and number of organ failures at ICU admission. Continuous variables were standardized to allow comparison of effect sizes. Odds ratios are plotted on a logarithmic scale

In-hospital mortality rates also differed significantly among clusters. Mortality was not significantly different in Cluster 1 (54%) and Cluster 2 (38%, p = 0.34), while Cluster 3 had the lowest mortality (9%, p = 0.007), although Clusters 2 and 3 had similar baseline severity (Fig. 3a).

Multivariate logistic regression further supported these findings, showing that in-hospital mortality was significantly higher for patients in Clusters 1 and 2 compared to Cluster 3, even after adjusting for admission severity (APACHE II score) and the number of organ failures (Fig. 3b). The final model included three predictors across four degrees of freedom, yielding an events-per-variable (EPV) ratio of 4.5 (Fig. 3b).

Internal validation using 1,000 bootstrap resamples confirmed the robustness of the model. The optimism-corrected c-index was 0.84, indicating good discriminative performance. The calibration slope was 0.76, and the maximum calibration error (Emax) was 0.077, reflecting adequate agreement between predicted and observed outcomes. The shrinkage-consistent g-index decreased from 3.3 (apparent) to 0.95 after correction, suggesting moderate optimism and acceptable model stability.

## Discussion

Our study identified three distinct clusters of critically ill patients with shock, spanning both septic and cardiogenic etiologies. These clusters were characterized by distinct metabolic signatures, varying degrees of clinical severity, and divergent outcomes, supporting the existence of biologically meaningful metabolic endotypes in acute shock.

Clusters 1 and 3 represented opposing metabolic archetypes. Cluster 1 included patients with the highest clinical severity and mortality (47%). This group was characterized by markedly elevated levels of biogenic amines, sugars, and sphingolipids, indicating a pronounced systemic response to stress and inflammation. Among biogenic amines, kynurenine, a downstream product of indoleamine-2,3-dioxygenase (IDO1) activity induced by IFN-γ, was notably elevated. Kynurenine has been implicated in oxidative stress [18], immunomodulation, and hemodynamic dysregulation through its vasodilatory effects via nitric oxide synthesis [19, 20]. In contrast, taurine levels were reduced, consistent with prior observations in critical illness [21], and may reflect depletion of cytoprotective osmolytes during mitochondrial dysfunction and oxidative stress [22].

The observed stress hyperglycemia in Cluster 1 suggests enhanced gluconeogenesis and glycogenolysis, potentially driven by insulin resistance [23, 24] and counter-regulatory hormones such as catecholamines, cortisol, and pro-inflammatory cytokines (e.g., IL-6, TNF-α) [25]. Sustained hyperglycemia contributes to mitochondrial overload and oxidative injury, both associated with adverse outcomes in the ICU setting [26].

Elevated sphingolipids, particularly ceramides, further underscore the inflammatory and injurious phenotype of Cluster 1. Ceramide accumulation via acid sphingomyelinase activation disrupts endothelial integrity, promotes apoptosis, and exacerbates vascular permeability [27–29]. Lower ceramide levels, by contrast, have been associated with improved endothelial function in sepsis [30]. Moreover, persistent sphingomyelinase activity and ceramide overload may promote immunoparalysis and metabolic shutdown, even in patients with preserved hemodynamics [31]. Together, these findings position Cluster 1 as a metabolically intense and injury-driven endotype, aligned with poor prognosis.

At the opposite end of the spectrum, Cluster 3 patients had the lowest clinical severity and mortality (8%) and displayed globally reduced metabolite levels. This metabolically"quiescent"profile suggests a stress-tolerant or well-adapted physiological response, suggesting preserved mitochondrial function and cellular homeostasis under critical illness [32].

In contrast, Cluster 2 represents a paradoxical phenotype. Although patients in this group exhibited clinical severity scores similar to those in Cluster 3, they experienced a markedly higher mortality rate (30%), comparable to that of the high-risk Cluster 1. Metabolically, Cluster 2 displayed a mixed profile: sphingolipids were elevated, suggesting ongoing systemic inflammation, yet biogenic amines and monosaccharides remained low, pointing to an absent or blunted systemic stress response. This atypical pattern may reflect a “hyporesponsive immunometabolic state,” a concept increasingly recognized in the literature, in which patients are unable to initiate an adequate metabolic defense due to insufficient mitochondrial and energetic reserves. Such a state has been linked to pre-existing immune exhaustion, low-grade chronic inflammation, aging, and comorbidities, factors that may compromise the ability to adapt to acute physiological insults [33–38]. Notably, Cluster 2 was the only group with elevated glycerophospholipids, which are central to membrane integrity and cellular signaling. This may reflect both adaptive and maladaptive processes. On one hand, increased glycerophospholipid biosynthesis has been described as an early compensatory mechanism to counteract oxidative stress and support membrane repair [39–41]. On the other hand, sustained elevation may lead to excessive lipid turnover and the generation of pro-inflammatory lysophospholipids—particularly via phospholipase A2 activation—exacerbating endothelial injury, leukocyte recruitment, and neutrophil extracellular traps (NET) formation [42]. This maladaptive lipid remodeling may overwhelm cellular repair mechanisms, leading to organ dysfunction and, ultimately, worse outcomes. When coupled with an insufficient metabolic stress response, evidenced by persistently low levels of biogenic amines and sugars, it may help explain the unexpectedly high mortality observed in Cluster 2, despite a deceptively mild clinical presentation.

Altogether, these findings suggest that responses to shock are not solely driven by clinical severity but are also shaped by individual biological variability. Pandey et al. demonstrated that underlying comorbidities, such as diabetes, chronic kidney disease, and cardiovascular dysfunction, can significantly alter the plasma metabolome, particularly in pathways involving amino acid and lipid metabolism [35, 43]. These intrinsic factors may impair stress adaptation, immune activation, and mitochondrial resilience. Additionally, sex-based metabolic differences in sepsis have been reported, with females showing higher concentrations of β-oxidation intermediates and lower levels of inflammatory metabolites compared to males, potentially affecting both disease progression and therapeutic responsiveness [44, 45].

Importantly, these individual host factors interact with the nature and extent of organ-specific dysfunction, which further modulates the metabolic phenotype. Different types of organ failure, including Acute Kidney Injury (AKI), acute and acute-on-chronic liver failure (ALF/ACLF), and acute respiratory distress syndrome (ARDS), exhibit distinct signatures across key metabolic classes: biogenic amines, sphingolipids, glycerophospholipids, and sugars.

In AKI, profound disruptions are observed in lipid metabolism, particularly affecting glycerophospholipids and sphingolipids. Spatial lipidomics revealed a regional increase in lysophosphatidylcholines (LysoPCs) such as LysoPC 16:0/0:0 and LysoPC 18:2 in both the renal cortex and medulla, indicating active membrane remodeling and damage. Conversely, sphingomyelin species (e.g., SM d18:0/16:0, d42:1) were significantly reduced in the renal cortex, suggesting impaired sphingolipid biosynthesis. These alterations were associated with enzymatic dysregulation involving reduced LPC acyltransferase and increased choline phosphotransferase activity, which may drive lipid toxicity and tubular injury. Regarding biogenic amines, experimental models showed elevated indoxyl sulfate—a known uremic toxin—early after ischemic injury, supporting its role as a marker of tubular stress and injury [46, 47].

In liver failure, a single targeted metabolomic study highlights significant alterations in lipid and energy metabolism. Patients with severe acute liver failure due to hepatitis E showed marked reductions in glycerophospholipids (e.g., choline, phosphocholine) and acylcarnitines, which are essential for mitochondrial function and immune competence. These deficits were associated with immunosuppression and worse clinical outcomes. The same study reported increased levels of conjugated bile acids and disruption of biogenic amines and glucose metabolism, consistent with hepatic failure and systemic stress [48].

In ARDS, profound disruptions were observed across several key metabolic classes. Glycerophospholipid metabolism was consistently altered, with significant reductions in circulating lysophosphatidylethanolamines (LysoPEs) and phosphatidylethanolamines (PE plasmalogens), particularly in non-survivors, reflecting membrane breakdown and impaired lipid remodeling. Sphingolipid metabolism was also disrupted, notably with decreased levels of sphingosine-1-phosphate (S1P), a lipid mediator essential for endothelial integrity; this reduction was most pronounced in indirect ARDS and associated with increased vascular permeability. While biogenic amines were not the primary focus, tryptophan metabolism—including downstream kynurenine pathway activity—emerged as significantly altered, indicating possible immune-metabolic dysregulation. Lastly, changes in glucose and energy metabolism were also significant, with pathways such as glycolysis and pyruvate metabolism contributing to the ARDS metabolic phenotype [49, 50].

These organ-specific metabolic signatures, when superimposed on individual host characteristics, contribute to the complex biological heterogeneity observed in critically ill patients. This complexity underscores the limitations of one-size-fits-all strategies in critical care. Given this biological heterogeneity, a personalized approach to critical illness becomes increasingly relevant. Previous studies have shown that metabolomic profiling can uncover distinct subgroups among patients with sepsis, with implications for both prognosis and treatment. Unsupervised clustering methods have revealed metabolic subphenotypes associated with systemic inflammation, organ failure, and mortality [18, 35, 40, 43, 44]. For example, Antcliffe et al. identified and externally validated three reproducible metabolic endotypes in septic shock using two independent randomized trial cohorts (LeoPARDS, n = 470; VANISH, n = 173) [40]. One of these phenotypes, characterized by persistently low lysophospholipid concentrations, was independently associated with significantly increased mortality (OR 3.66 [1.88–7.20], p < 0.001 in LeoPARDS; OR 4.13 [1.17–15.61], p = 0.03 in VANISH). These profiles were not only prognostically relevant but also dynamic over time, closely tracking with the evolution of organ dysfunction. This highlights the potential of metabolomic clustering not only as a static risk stratification tool at admission but also as a longitudinal monitoring strategy during the ICU stay. Similarly, Rogers et al. described lipid-driven subtypes correlated with divergent trajectories and organ dysfunction patterns [18]. These findings support the concept of temporal metabolic trajectories in sepsis, where patients may remain in or transition between phenotypes with distinct clinical implications [40].

Taken together, our findings and previous studies underscore that metabolic phenotyping captures dimensions of individual biological response that extend beyond clinical severity or the extent of organ failure. These insights support its integration into future precision medicine strategies in critical care, where it could help identify high-risk endotypes, guide personalized interventions, and enable longitudinal monitoring of dynamic biological state.

Several limitations of this study must be acknowledged. First, the relatively small sample size (n = 60) limits the generalizability of our findings. Although our unsupervised clustering approach demonstrated robust internal stability and biological coherence, external validation in an independent, multicenter cohort will be essential to confirm the reproducibility of the identified metabolic phenotypes. Second, the observational and exploratory design of this study precludes causal inference between metabolic profiles and clinical outcomes. While our primary goal was to identify biologically meaningful patient subgroups, further mechanistic studies are needed to elucidate the molecular pathways underpinning each phenotype and to evaluate their therapeutic relevance. Third, metabolite concentrations can vary substantially across studies [3] due to technical, biological, and analytical differences. Our aim, however, was not to focus on individual metabolite levels but rather to identify broader metabolic signatures and patterns associated with prognosis. Fourth, although metabolomics offers deep biological insights, its complexity and cost currently limit widespread clinical use. A key translational step will be the development of predictive models that infer metabolic phenotype from standard clinical and laboratory data available at ICU admission. Such models could enable early risk stratification, guide phenotype-targeted interventions, and allow retrospective reanalysis of existing ICU cohorts.

Despite these limitations, our study offers important insights into the metabolic heterogeneity of shock and provides a foundation for future precision approaches in critical care.

## Conclusion

In this study, we identified three metabolically and clinically distinct phenotypes among critically ill patients with shock, highlighting the heterogeneity of host responses beyond conventional severity scores or shock etiology. These metabolic profiles, shaped by both individual biological predispositions and organ-specific dysfunctions, were strongly associated with outcomes, including a clinically deceptive high-risk subgroup. Our findings reinforce the potential of metabolomic clustering as a framework for personalized risk stratification in the ICU. Future work should focus on validating these phenotypes in independent cohorts, unraveling their mechanistic underpinnings, and integrating them into predictive models that bridge complex biology with bedside applicability.

## Supplementary Information

Additional file 1. Figure 1. Distribution of Jaccard similarity indices across 1000 bootstrap iterations, assessing the stability of cluster assignments. Each bar represents the frequency of Jaccard scores between resampled and original clustering results. The dashed vertical line indicates the mean Jaccard index. Figure 2. Severity Profiles of Patients at Inclusion Box plots illustrating the levels of severity variables at inclusion across clusters. Table 1. List of the measurable metabolites using the Biocrates Absolute IDQ p180 kit. Table 2. Pairwise comparisons of baseline characteristics across metabolomic clusters

## Data Availability

The datasets generated and/or analysed during the current study are available from the corresponding author on reasonable request.
