# Supplementary material for: Metabolomic stratification of shock: pathophysiological insights for personalized critical care
Source: Ann Intensive Care. 2025 Jul 31;15:109. doi: 10.1186/s13613-025-01532-1 (PMC12314137; doi:10.1186/s13613-025-01532-1)

Supplementary Table 1

List of the measurable metabolites using the **Biocrates Absolute IDQ p180 kit**.

METABOLITE CLASS	N	RETAINED FOR ANALYSIS	NOT RETAINED FOR ANALYSIS
**AMINO ACIDS**	21	alanine, arginine, asparagine, citrulline, glutamine, glutamate, glycine, histidine, isoleucine, leucine, lysine, methionine, ornithine, phenylalanine, proline, serine, threonine, tryptophan, tyrosine, valine	aspartate
**CARNITINE**	1	C0	-
**ACYLCARNITINE**	39	C2, C3,	C3:1, C3-OH, C4, C4:1, C4-OH, C5, C5:1, C5:1-DC, C5-DC, C5-M-DC, C5-OH, C6, C6:1, C7-DC, C8, C9, C10, C10:1, C10:2, C12, C12-DC, C14, C14:1, C14:1-OH, C14:2, C14:2-OH, C16, C16:1, C16:1-OH, C16:2, C16:2-OH, C16-OH, C18, C18:1, C18:1-OH, C18:2, C12:1
**BIOGENIC AMINES**	19	Acetylornithine, asymmetric dimethylarginine, total dimethylarginine, alpha-aminoadipic acid, creatinine, kynurenine, methionine sulfoxide, hydroxyproline, spermidine, taurine	carnosine, histamine, nitrotyrosine, phenylethylamine, putrescine, sarcosine, serotonin, spermine
**GLYCEROPHOSPHOLIPIDS:** **LYSO-PHOSPHATIDYLCHOLINES**	14	lysoPC.a. C16:0, C16:1, C17:0, C18:0, C18:1, C18:2, C20:3, C20:4, C26:0, C26:1, C28:0, C28:1, C24:0	lysoPC.a. C14:0
**GLYCEROPHOSPHOLIPIDS:** **DIACYL-PHOSPHATIDYLCHOLINES**	38	PC.aa. C24:0, 28:1, C30:0, C30:2, C32:0, C32:1, C32:3, C34:1, C32:2, C34.2, C34:3, C34:4, C36:0, C36:1, C36:2, C36:3, C36:4, C36:5, C36:6, C38:0, C38:1, C38:3, C38:4, C38:5, C38:6, C40:3, C40:4, C40:5, C40:6, C42:0, C42:1, C42:2, C42:4, C42:5	PC.aa. C26:0, C42:6, C40:1, C40:2,
**GLYCEROPHOSPHOLIPIDS:** **ACYL-ALKYL-PHOSPHATIDYLCHOLINE**	38	PC.ae. C30:1, C30:2, C32:1, C32:2, C34:0, C34:1, C34:2, C34:3, C36:0, C36:1, C36:2, C36:3, C36:4, C36:5, C38:0, C38:1, C38:2, C38:3, C38:4, C38:5, C38:6, C40:1, C40:2, C40:3, C40:4, C40:5, C40:6, C42:2, C42:3, C42:4, C42:5, C44:3, C44:4, C44:5, C44:6	PC.ae. C30:0, C42:0, C42:1
**SPHINGOLIPIDS**	15	SM (OH) C14:1, SM C16:0, SM (OH) C16:1, SM C16:1, SM C18:0, SM C18:1, SM C20:2, SM (OH) C22:1, SM (OH) C22:2, SM C24:0, SM C24:1, SM (OH) C24:1, SM C26:0, SM C26:1	SM C22:3
**MONOSACCHARIDE**	1	sugar	-
**TOTAL**	186	130	56

Supplementary Table 2

Pairwise comparisons of baseline characteristics across metabolomic clusters

	Cluster 1 vs Cluster 2	Cluster 1 vs Cluster 3	Cluster 2 vs Cluster 3
**ADMISSION CAUSE**	0.472	0.760	0.723
**DEMOGRAPHICS AND UNDERLYING DISEASES**			
Sex, male, n (%)	0.716	0.494	0.235
Age, mean (IQR)	0.435	0.273	0.553
Arterial hypertension, n (%)	0.315	0.772	0.501
Diabetes Mellitus, n (%)	<0.001	<0.001	>0.999
Coronary artery disease, n (%)	0.685	>0.999	0.682
Other cardiovascular disease, n (%)	0.278	0.188	>0.999
Chronic lung disease, n (%)	0.691	0.701	0.225
Chronic kidney disease, n (%)	0.394	0.416	>0.999
Chronic liver failure, n (%)	0.538	>0.999	0.525
**CHARACTERISTICS AT ICU ADMISSION**			-
Anuria and/or RRT, n (%)	>0.999	0.489	0.361
Systolic arterial blood pressure (mmHg), mean (IQR)	>0.999	0.741	0.843
Diastolic arterial blood pressure (mmHg), mean (IQR)	0.911	0.601	0.597
Mean arterial blood pressure (mmHg), mean (IQR)	>0.999	0.543	0.655
Heart rate (bpm), mean (IQR)	0.577	0.790	0.908
Creatinine (mg/dL), mean (IQR)	0.219	0.693	0.186
Na^+ (mmol/L), mean (IQR)	0.936	0.923	0.766
K^+ (mmol/L), mean (IQR)	0.702	0.337	0.767
Cl^- (mmol/L), mean (IQR)	0.185	0.480	0.298
Lactate, mean (IQR)	0.005	0.190	<0.001
pH, mean (IQR)	0.077	0.156	0.015
PaO2 (mmHg), mean (IQR)	0.279	0.454	0.051
PaCO2 (mmHg), mean (IQR)	0.265	0.149	0.008
$HCO3^-$ (mmol/L), mean (IQR)	>0.999	0.596	0.572
Base Excess (mmol/L), mean (IQR)	0.127	0.921	0.073
SOFA score, mean (IQR)	0.821	0.049	0.035
APACHE II score, mean (IQR)	0.084	0.149	0.023
**OUTCOMES**			
ICU stay (d), mean (IQR)	0.655	0.257	0.727
Hospital stay (d), mean (IQR)	0.774	0.306	0.644
ICU mortality, n (%)	>0.999	0.072	0.073
Hospital mortality, n (%)	0.489	0.036	0.005

Supplementary Figure 1

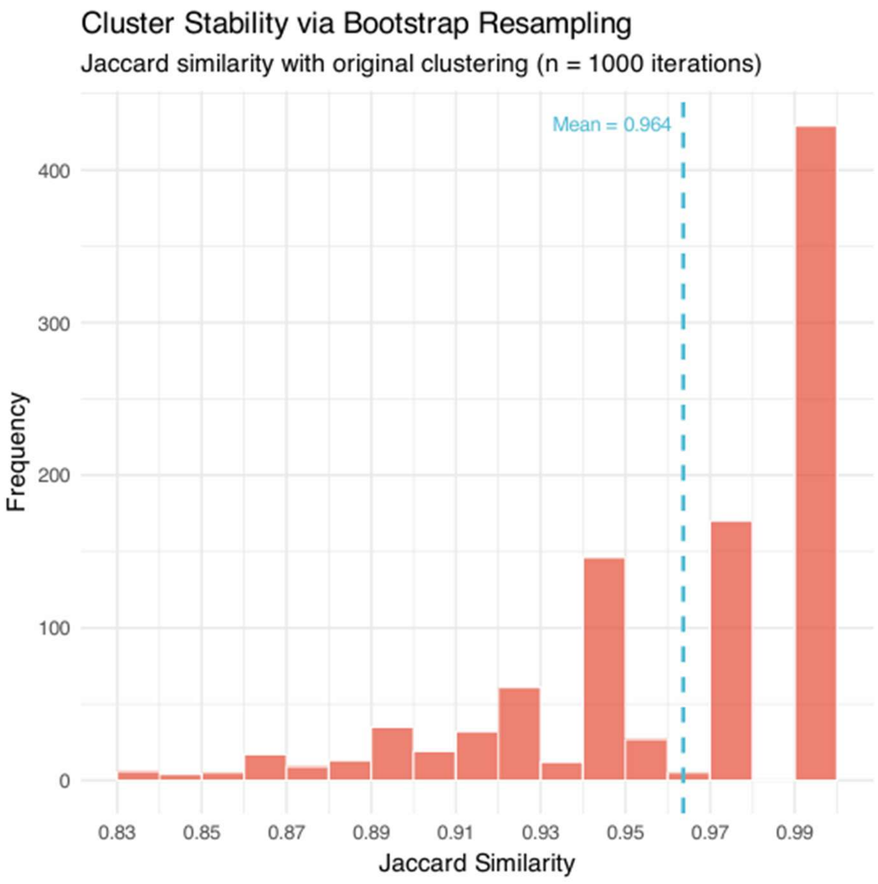

Supplementary Figure 2

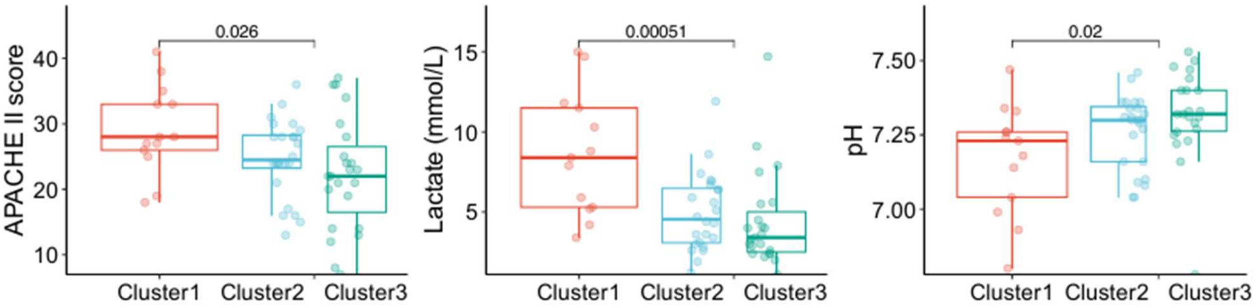

Supplement: Supplementary file 1 — Additional file 1. Figure 1. Distribution of Jaccard similarity indices across 1000 bootstrap iterations, assessing the stability of cluster assignments. Each bar represents the frequency of Jaccard scores between resampled and original clustering results. The dashed vertical line indicates the mean Jaccard index. Figure 2. Severity Profiles of Patients at Inclusion Box plots illustrating the levels of severity variables at inclusion across clusters. Table 1. List of the measurable metabolites using the Biocrates Absolute IDQ p180 kit. Table 2. Pairwise comparisons of baseline characteristics across metabolomic clusters [file 13613_2025_1532_MOESM1_ESM.pdf]
